# Integrative Long Non-Coding RNA Analysis and Recurrence Prediction in Cervical Cancer Using a Recurrent Neural Network

**DOI:** 10.3390/diagnostics15222848

**Published:** 2025-11-10

**Authors:** Geeitha Senthilkumar, Renuka Pitchaimuthu, Prabu Sankar Panneerselvam, Rama Prasath Alagarswamy, Seshathiri Dhanasekaran

**Affiliations:** 1 Department of Information Technology, M. Kumarasamy College of Engineering, Thalavapalayam, Karur 639113, Tamil Nadu, India; geethas.it@mkce.ac.in (G.S.); renukap.it@mkce.ac.in (R.P.); 2 Shanmuga Hospital, Salem 636007, Tamil Nadu, India; drprabusankar@smrft.org; 3 Department of Computer Applications, SRM Institute of Science and Technology, Trichy 620015, Tamil Nadu, India; ramaprasath.a@ist.srmtrichy.edu.in; 4 Department of Computer Science, UiT The Arctic University of Norway, 9037 Tromsø, Norway

**Keywords:** prognosis, long non-coding RNA, biomarker, recurrent neural network, recurrent cervical cancer

## Abstract

**Background:** Recurrent cervical cancer is one of the most defining threats to patient longevity, underscoring the need for prognostic models to identify high-risk patients. **Objectives:** The aim of the study is to integrate clinical data with the GSE44001 Dataset to identify key risk factors associated with the recurrence of cervical cancer. Patients are stratified into high-, moderate-, and low-risk groups using selected clinical and molecular features. Identifying a long non-coding RNA (lncRNA) gene signature associated with recurrent cervical cancer. **Methods:** From the total data collected, 138 recurrent cervical cancer patients were identified. GSE44001 Dataset is downloaded from the NCBI GEO Database. When using the GENCODE Annotation tool, the long non-coding RNA is filtered. The dataset is then linked with filtered long non-coding RNA. The Least Absolute Shrinkage Selection Operator (LASSO) is employed to find attributes in gene expression analysis. Risk factors of recurrent cervical cancer are identified. Risk value is assigned to each individual based on the selected lncRNAs and the corresponding overfitting coefficients. **Result:** The RNN Long Short-Term Memory model demonstrates a prognostic value, where high-risk patients experience a shorter duration of recurrence-free survival (*p* < 0.05). Individuals with a recurrence of cervical carcinoma, a progressive disease, were associated with the ATXN8OS marker, the C5orf60 indicator, and the INE1 index gene. In contrast, patients diagnosed at earlier stages are aligned with the KCNQ1DN marker, LOH12CR2 gauge, RFPL1S value, and KCNQ1OT1 indicator. Patients in moderate stages were primarily associated with the EMX2OS score. **Conclusions:** The research findings demonstrate that the nine-lncRNA signature, when combined with deep learning, offers a powerful approach for recurrence risk stratification in cervical cancer.

## 1. Introduction

Cervical cancer is the leading cause of cancer-related mortality among women worldwide. There are many advances in treatment, but recurrence rates remain significant and represent a hurdle for long-term survival. Identifying accurate risk factors of recurrence is important. Clinical parameters, such as tumor stage, lymph node metastasis, and tumor diameter, are associated with the risk of recurrence. Many studies have shown that long non-coding RNAs (lncRNAs) play a vital role in tumor progression and prognosis. Long non-coding RNA, such as HOTAIR and MALAT1, has been linked to cervical cancer progression, but the predictive potential of lncRNA signatures for recurrence has received limited attention. Gaps in the existing literature include that in prior studies, the investigations were conducted with either clinical factors or molecular features independently, without combining both sources of data in an integrated forecasting model. This research aims to integrate real-world clinical data with transcriptomic data (GSE44001) to identify key recurrence-related factors and construct a deep learning based predictive model. By combining clinical and molecular features, a clinically useful tool will be developed for assessing recurrence risk in cervical cancer patients in the future. Patients are stratified into high-, moderate-, and low-risk groups using selected clinical and molecular features. Identifying long non-coding RNA (lncRNA) gene signature associated with recurrence in cervical cancer.

Preventing cervical carcinoma is possible with early detection, as it is the most prevalent cancer in women [1]. Cervical cancer causes many deaths in women [2]. Recurrence of cervical cancer is the development of a tumor in a local area or distant metastasis within six months following the completion of initial treatment. Recurrence of cervical carcinoma commonly occurs in two-thirds of patients who have undergone cervical carcinoma treatment within the two years. Computed Tomography and Magnetic resonance imaging are the main techniques that identify recurrent cervical cancer [3]. Forecasting the recurrence of cervical cancer is a challenging issue. An AI-powered neural network method addresses these issues [4]. Between 1994 and 2004, the MD Anderson Carcinoma Center treated 28 individuals who underwent pelvic exoneration for recurrent cervical carcinoma [5].

Doctors from 21 medical centers in Norway surveyed 58 cervical cancer patients between 2012 and 2016. Medical and self-reported characteristics were collected using a standardized survey [6]. The risk of recurrence and mortality is increased in patients with tumor size, advanced FIGO stages, and lymph node involvement. As tumor size increases, the likelihood of recurrence also increases. Due to these results, Gynecologists schedule regular patient appointments and determine the tumor possibility for patients with cervical cancer [7]. Medical professionals identify recurrent cervical cancer by evaluating the risk factors with their clinical expertise. Scientific study and practical application are attempting to identify significant risk factors for recurrent [8]. Clinicians categorize the patients by prognosis, assign them to treatment studies, and perform precise and repeated evaluations to improve results [9]. The study employs K-means clustering, principal component analysis, and multivariate Cox survival analysis to assess the variables that influence recurrence prediction. The study evaluates eight distinct machine learning methods using logistic and Cox models [10]. By the optimization of some variables, Cox regression analysis demonstrates that treatments given, clinical phase, and premature delivery were all substantially linked to the recurrence of cervical cancer [11].

Patients at stage 1A1 did not want exceptional medical follow-up in cervical cancer treatment. This conclusion is supported by the fact that, during the previous nine years, there were only eight recurrences and one death among the 510 patients in this cohort [12]. Four long non-coding RNAs (lncRNAs) were significantly linked to a lower recurrence-free outcome in cervical cancer using Cox regression analysis. MIR22HG is a predictive biomarker for cervical carcinoma [13]. This research examines the relationship between diagnostic techniques and their effectiveness in relation to survival rates across various clinicopathological features [14]. Web-based research for recurrent cervical cancer treatment and care was conducted in the Medline and CancerLit databases. Randomized research investigations have been given priority in the collection and analysis of all pertinent information [15]. The purpose of the research is to figure out the clinical value of the lncRNA SPRY4-IT1 in the advancement of cervical carcinoma, as well as assess its expression level.

According to research, lncRNA SPRY4-IT1 is a novel molecule associated with the progression of cervical carcinoma. As such, it could be a helpful target for therapy, as well as a potential prognostic indicator [16]. A recurrent neural network (RNN) is employed to process information related to gene expression, as described in the research. LSTM and GRU, two varieties of RNNs, were examined. A proposed method for improving the RNN’s design and hyperparameter scores calculates the sample precision for classification, as well as the F1 score. This parameter enables assessments of sample distribution into the appropriate classes [17].

## 2. Related Works

Multiple bioinformatics approaches are used to detect 143 Differentially expressed Genes [DEG] related to cervical carcinoma and show that these genes contribute to cervical cancer development [18]. Grated Recurrent Unit-based Recurrent Neural Network forecasts the clinical outcomes, showing its strength on sequence data and retains the past information [19]. Researchers employed data mining through the NCBI Gene Expression Omnibus to ascertain the medical importance of certain DEGs in cervical carcinoma in women [20]. A nine-lncRNA signature achieved enhanced predictive precision than FIGO Stage [21].

To investigate the survival experience of cervical cancer patients who experience recurrence, a recurrence model based on the nomogram is used, analyzing the signatures of long non-coding RNA [22]. Early immunological status assessment of cervical cancer patients is facilitated by a prognostic framework based on Lymph node metastasis-relevant long non-coding RNAs (lncRNAs), as outlined in the HSIC Model [23]. A total of 289 RNA sequence data and related clinical information were acquired. Two of the forty-nine lncRNAs that we found to be differentially expressed were linked to prolonged life for patients with cervical cancer. Collectively, both of these lncRNAs (RAS4CP and ILF3-AS1) have been identified to form a single predictive characteristic.

In contrast, women with low-risk cervical cancer had an improved prognosis and saw a significant correlation in overall survival (*p* < 0.001). Subsequent investigation revealed that using the two-lncRNA expression profile together could potentially be utilized as a stand-alone indicator of the cervical cancer patients’ prognosis [24]. Identifying the risk factors of recurrent cervical cancer is essential for treatment management and improved outcomes [25]. A prognostic scoring model is developed to predict patient outcomes through integrative analyses that identify ion channel-related long non-coding RNAs (lncRNAs) [26]. The recently discovered functions of lncRNAs in cervical carcinoma, namely in relation to metabolic restructuring, HPV control, therapy obstruction, cancer metastases, and cancer development. There is a vast potential for lncRNAs to be indicators for the detection of cervical carcinoma [27].

If clinicians knew the prognosis of recurrence, then patients would receive therapy earlier. A hybrid approach is recommended to address this issue. Features are extracted via transfer learning, which is then integrated with conventional machine learning to assess and ascertain a patient’s likelihood of metastases and recurrence [28]. The suggested method performs multi-label categorization using the patient’s medical visit data by extending an extended short-term memory system with two distinct approaches [29]. Language, series of times, and voice are examples of sequential data that may be handled by neural network architectures such as the recurrent neural network (RNN). An RNN’s fundamental idea is that it retains links to previous states, which allows the algorithm to retain knowledge about aspects of the preceding sequences [30,31,32].

## 3. Materials and Methods

Clinical information is collected from patients with cervical cancer undergoing therapy at Shanmuga Hospital, Salem. The data consists of complete demographic details of patients with cervical cancer, containing several variables that might affect mortality and recurrence.

**Figure 1:**
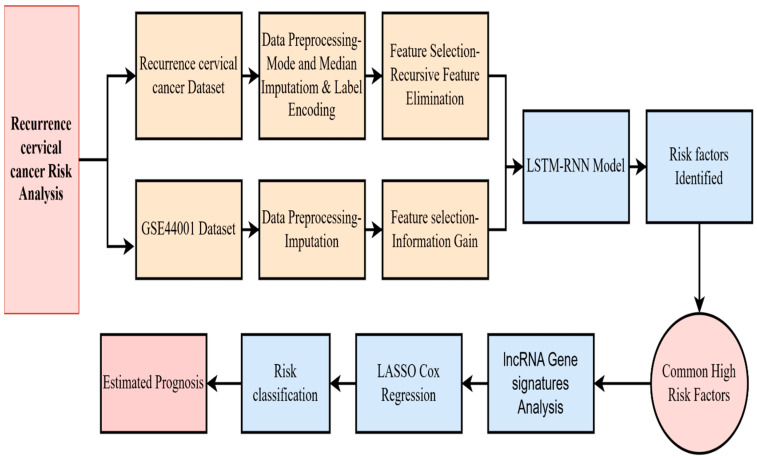
Proposed research method.

The research analyzed a total of 739 cervical cancer patients with 29 clinical and pathological features, in which 138 patients experienced recurrence during continuous follow-up [33]. The proposed architecture, illustrated in Figure 1, involves preprocessing the dataset by imputing missing values using mean and mode, encoding categorical variables through label encoding, and normalizing the data using MinMax scaling. The most important features are selected using Recursive Feature Elimination. A Long Short-Term Memory (LSTM) recurrent neural network is deployed to find key risk factors associated with recurrence. Clinical high-risk factors are tumor stage, lymph node metastasis, tumor size, PV/PR examination, and disease-free survival rate. To enhance the analysis, the publicly available GSE44001 dataset is acquired from the NCBI GEO. The Information Gain method is used for feature selection. The GENCODE Annotation tool is used to filter out the long non-coding RNA [34].

The samples are matched to the original dataset based on stage and recurrence status. Patients are classified into high, moderate, and low-risk groups using the selected clinical and molecular features. Significant long non-coding RNAs (lncRNAs) were identified through LASSO regression, and individual risk scores were calculated using the corresponding LASSO coefficients to evaluate recurrence risk for each patient [35]. Thus, Table 1 below shows the recurrence of some categories of cervical cancer patient details.

**Figure 2:**
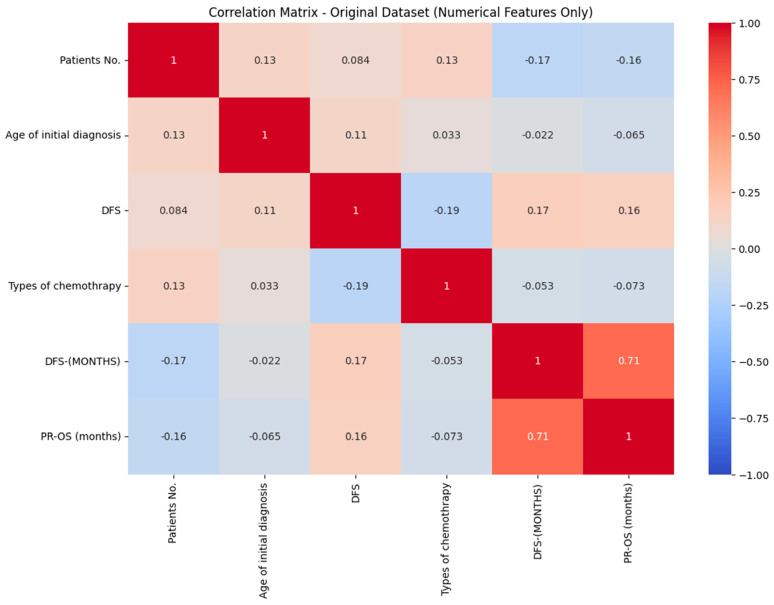
Correlation matrix of RCC.

The correlation coefficient is 0.71 for disease-free survival, and progression-free overall survival has a strong positive correlation. This indicates that prolonged disease-free survival is linked with extended overall survival in patients. A negative correlation indicates a weaker relationship between the variables, as illustrated in Figure 2.

Table 2 represents the statistical table for numeric values in the dataset. This table presents the mean and median calculations, along with the standard deviation, for numeric columns to extract information from the dataset.

### 3.1. Data Preprocessing

Preprocessing is an important stage to ensure the information is processed and developed for Neural Network algorithms to function effectively. To handle the missing values in categorical features, the mode is used to replace the value. For numerical values, the median technique is used [36].

Handling missing values in a dataset is crucial for creating a well-structured dataset and obtaining consistent results. For a categorical attribute Y with values ${(y}_{1},y_{2},\ldots ,y_{n})$, the values that appear most frequently in a dataset are replaced in Y. In Equation (1), the mode is defined as the element y within the set Y, that has the highest frequency of occurrence.

(1) $$Mode\left(Y\right)=y_{max}$$

$y_{max}—value\;of\;y\;that\;appeared\;a\;maximum\;time$.

Median techniques are used for numerical data and are effective against the outliers. In Equation (2), the median is the middle value of the dataset if it is sorted in ascending order. If m is an odd number, then the median is the middle value of the dataset. If the dataset has an even number of elements, then the median is the average of the two middle elements.

(2) $$Median\left(Z\right)=\left\{\begin{array}{c} z_{(m+1)/2}\;\;\;\;\;\;\;\;if\;m\;is\;odd\\ \;\;\frac{z_{\frac{m}{2}}+z_{\left(\frac{m}{2}\right)+1}}{2}\;\;\;\;\;\;\;if\;m\;is\;even \end{array}\right\}$$

$Z={(z}_{1},z_{2},\ldots ,z_{m)}\;in\;sorted\;data$.

m—Number of elements.

Table 3 detailing the proportion of missing data per feature is included above. Label encoding is used to transform all categories which is not numerical variables into numeric depictions. A dataset consisting of categorical features, such as sex, different diagnosis categories, and imaging techniques, needs to be converted into a numerical form for further analysis. For a label encoding method, each feature is assigned to a unique numerical value.

In Equations (3) and (4), it means that $y=g_{i}$, then $f\left(y\right)=i$. For example, it means that if Stage (one of the attributes in a dataset) has four categories, the encoding is performed. Stage 1 is represented as 0, Stage 2 is represented as 1 and so on. f(y) produces a numerical value where y is a categorical variable.

For a categorical feature y having categories $\left\{g_{1},g_{2}\ldots ,g_{n}\right\}$

(3) $$f:y\to z,\;where\;f\left(g_{i}\right)=i(i.e\;g_{1}\to 0,g_{2}\to 1,g_{3}\to 2\ldots ,g_{4}\to n-1)$$

Mathematically:

(4) $$f\left(y\right)=i;where\;y\;=\;g_{i}\;and\;i\epsilon \left(0,1,\ldots ,n-1\right)$$

Machine learning methods, such as Random Forest, require numerical inputs; therefore, categorical parameters need to be converted into numerical values.

The proposed model utilizes MinMaxScaler to scale each feature to a range of 0 to 1, as shown in Equation (5). Different features in the dataset have different ranges, but the LSTM RNN model works well with normalization. The MinMax Scaler is used to scale each attribute z in the dataset to a range of 0 to 1.

(5) $$Z_{min-max}=\frac{z-z_{min}}{z_{max}-z_{min}}$$

z—original attribute in a data.

$z_{min}\;and\;z_{max}\;are\;the\;minimum\;and\;maximum\;values\;of\;that\;attribute$.

$Z_{min-max}—The\;normalised\;value$.

LSTM can capture intricate, non-linear connections in high-dimensional long non-coding RNA (lncRNA) expression and clinical data. LSTM networks provide a distinct benefit over conventional models. In cases where the input is not sequential, its gated design is allowed. As a result, when compared to conventional analytical methods, LSTMs are best in forecasting the recurrence of cervical cancer.

The Neural Network model, particularly the Long Short-Term Memory model (LSTM), is dependent on the size of its input, which is crucial. MinMaxScaler makes sure that each attribute makes an equal contribution. These outcomes show the prognostic value of the detected lncRNA and clinical variables, and are the primary sources of the predictive model.

### 3.2. Feature Selection

Feature selection is for selecting important attributes in a given data set that help the algorithm concentrate on the relevant data and reduce the chance of overfitting. A Random Forest Classifier is used as a foundational framework for Recursive Feature Elimination (RFE) [37]. RFE recursively adapts the framework by eliminating fewer significant features until the necessary number of characteristics is obtained. RFE enables the framework to maintain the most essential features while repeatedly eliminating the less significant features. This lessens the level of complexity of the model, which might enhance generalization.

The dataset consists of 29 features $Z={\{z}_{1},z_{2},\ldots ,z_{29}\}$. To find the important attributes k, where k < 29, and to shortlist the important attributes to enhance the model’s performance.

$$z_{t}=set\;of\;features\;at\;step\;t$$

$$I_{t}=set\;of\;Feature\;Importance$$

Train $N\;on\;z_{t}:$

(6) $$\mathrm{Calculate},\;{\;\;I}_{t}=\{I_{t1,}I_{t2,\ldots ,}I_{t,\left|Z_{t}\right|}\}$$

In Equation (6), the model assigns an importance score to each feature, and these scores are collected as $I_{t}$.

(7) $$\mathrm{To}\;\mathrm{identify}{,\;\;Z}_{min,t}=\arg{min}_{i}\left(I_{t},i\right)$$

Equation (7) is to identify the feature with the minimum importance score in the step $I_{t}$:

(8) $$\mathrm{Set},Z_{t+1}=\frac{Z_{t}}{\left\{Z_{min,}t\right\}}$$

Equation (8) finds the feature with the least important score.

The recurrence will be stopped once $|z_{t}|\;=\;k$, considering k as a crucial feature, Random Forest is capable of accurately capturing the relevance of features. It is vital for feature selection. Features that are selected as important include total full-term pregnancies, PV/PR Examination, history, largest diameter (in cm), lymph node metastasis, histological type, treatment type, stage, imaging, and disease-free survival rate (in months). These features are used to train the model.

### 3.3. RNN LSTM

**Figure 3:**
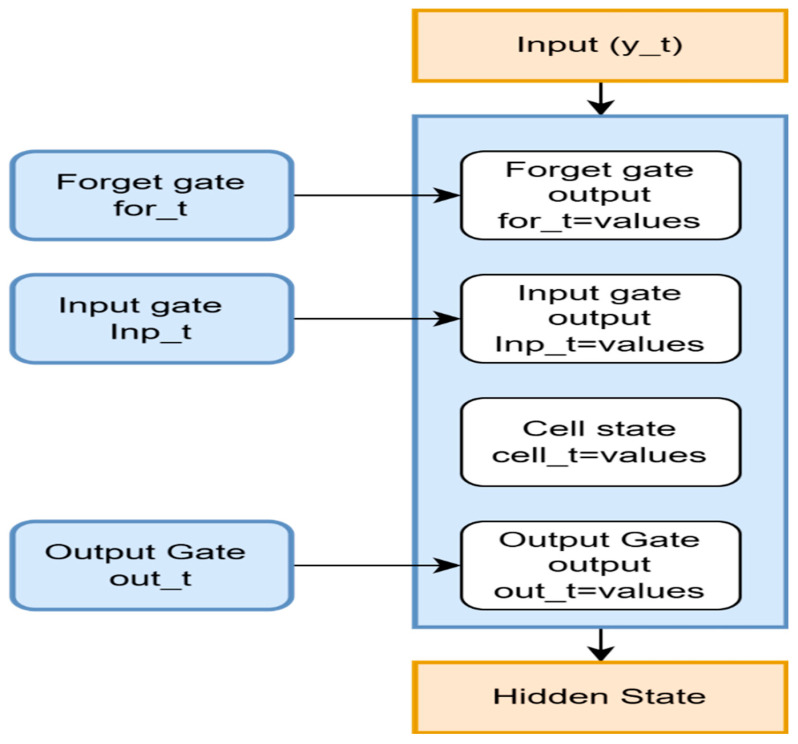
A recurrent neural network LSTM to find the relevant features.

A recurrent neural network model is developed, utilizing a long short-term memory (LSTM) model with sequential data [38]. Through the selected attributes, the LSTM recurrent neural network is used to analyze sequential patterns. To accurately predict the recurrence of cervical cancer, the LSTM is used to find the subset of relevant features. LSTM determines if an individual is at significant risk of recurrence by analyzing similarities in periodic data of clinical factors over time, as shown in Figure 3, if the chosen characteristics contain such information.

LSTM is excellent for handling information with temporal dependence. LSTM performs well with organized and sequential information; it was employed in this dataset, which is not quite a time series. The structure consisted of two layers of LSTM. The initial layer consisted of 64 units, while the subsequent layer comprised 32 units. To avoid overfitting, a dropout layer is added after every LSTM layer. This forces the framework to avoid overfitting and improve generalization by randomly dropping neurons throughout training. A dropout rate of 0.3 is employed. The LSTM layers underwent L2 regularization to penalize large values and prevent further overfitting [39]. The mathematical framework is kept from getting overly dependent on any particular characteristic process. Since this involves a problem with binary classification, such as forecasting the recurrence of cervical carcinoma, a sigmoid activation function is utilized in the outcome layer. Algorithm 1 is the Identification of relevant features. The Adam optimizer is used for effective learning; the model is constructed using binary cross-entropy as the loss function. By analyzing performance metrics, accuracy is found.

**Algorithm 1.** Identification of Relevant FeaturesFirst stage: Preparing the Dataset and Setting Up Hyperparameters Step 1: Use the matrix method for displaying the expression of genes in the dataset.
(9)$$D={{(d}_{mn})}_{i*j}$$
i = No. of patient samples. j = The quantity of chosen lncRNA genes used as input characteristics.
${(d}_{mn})—Expression\;of\;gene\;n\;in\;patient\;m$. Step 2: Initialization of Hyperparameters Configure LSTM network-specific hyperparameters. One layer of LSTM at first, and up to three layers at most.
Initial value :k=30 Neurons
$$Range\;:k_{min}=30\;to\;k_{max}=80\;\;hence\;inceremnenting\;∆k=5$$
Step 3: Describe the activation processes. Use tanh activation in the LSTM’s internal processing. For the binary category of recurrence condition, recurrence versus non-recur rence employs sigmoid activation. Step 4: Splitting of Data.
$$E_{train}—70\%\;data\;for\;LSTM\;Model$$
$$E_{test}—30\%\;for\;final\;evaluation$$
2nd Stage: Developing LSTM Models using Hyperparameter Adjustment Step 5: The LSTM unit performs subsequent calculations at every step based on the gene expression pattern of each sample. Step 6: Forget gate: The forget gate regulates which data derived from the prior cell state should be kept.
(10)$$g_{t}=\sigma \left(U_{f}*\left[g_{t-1},y_{t}\right]+a_{f}\right)$$
$g_{t}—Forget\;gate\;output$.
$U_{f}$&$a_{f}—Forget\;gate\;Weight\;matrix\;and\;Bias\;vector$. $g_{t-1}—Previous\;hidden\;state$. $y_{t}—input$. Step 7: Input Gate: Choose which $y_{t}$ data should be incorporated into the:
(11)$$Cell\;state=\sigma \left(U_{i}*{[g}_{t-1},y_{t}\right]+a_{i})$$
(12)$${\hat{C}}_{t}=tanh(U_{c}*\left[g_{t-1},y_{t}\right]+a_{c})$$
$y_{t}—Input\;Gate\;Activation$.
${\hat{C}}_{t}—candidate\;cell\;state$.
$g_{t-1}—Previous\;hidden\;state$.
$U_{i},\;a_{i}$,$U_{c}$,$a_{c}—Respective\;weight\;and\;Biases$ Step 8: Cell state update-Improves the cell state through the combination of data from the past and present.
$$C_{t}=Cell\;state\;Update$$
(13)$$C_{t}=d_{t}*C_{t-1}+j_{t}*{\hat{C}}_{t}$$
$d_{t}—forget\;gate\;output$. $C_{t-1}—previous\;cell\;state$. $j_{t}—input\;gate\;output$. Step 9: Output gate—Uses the modified cell status for identifying the subsequent concealed state $g_{t}.$
(14)$$O_{t}=\sigma (U_{o}*\left[g_{t-1},y_{t}\right]*a_{o})$$
$O_{t}—output\;gate\;activation$. σ—sigmoid function. $U_{o}—weight\;matrix\;for\;output\;gate$. $g_{t-1},y_{t}—previous\;hidden\;state\;and\;current\;input$. $a_{o}—bias\;term$.
(15)$$g_{t}=O_{t}*tanh(C_{t})$$
Step 10: Output layer: The output layer receives the last concealed state $Z_{t},$ from the last LSTM cell.
(16)$${\;Z}_{t}=\sigma \left(U_{g_{0}}*g_{t}+a_{g_{0}}\right)$$
$Z_{t}—Probability\;of\;recurrence$.
σ—Activation function. $U_{g_{0}}—weight\;matrix$. $g_{t}—Hidden\;state$. Step 11: To track convergence and prevent overfitting, the LSTM model is trained with *E_Train_* and compute accuracy, F1 score, and loss. Step 12: Hyperparameter adjustment is made until the *k_max_ value reaches* 30.
Step 13: After determining the ideal arrangement, test the system on $E_{Test}$, and estimate final indicators, such as F1 score, accuracy, and ROC-AUC. 3rd Stage: Result analysis and optimal configuration.

A layered LSTM 10-fold cross-validation grid search method has been applied to adjust the LSTM classifier’s hyperparameters. The original single-layer LSTM, featuring 30 neurons, is designed for tuning, and the number of neurons has continuously increased in increments of ∆*k* = 5 until the maximum value of 80 neurons per layer was reached. In addition to differences in the number of batches, learning rates, and dropout rates, designs comprising one to three LSTM layers are assessed. The mean of each cross-validated AUC was the primary criterion chosen for sorting the suggested models.

The selected feature is given as input to an LSTM Model after normalization. These characteristics enable the forget gate to recall essential data, such as dangerous signs, by teaching it only to recall specific details of the prior state. The gate that receives input determines the level of additional data that must be sent to the cell’s state. Forget and input gates are activated to modify the Cell State [40]. It integrates data with the current input characteristics and the previously stored information from the prior stage. This Research employs LSTM because of its ability to capture complex, long-range dependencies among features. The integrated dataset of lncRNA expression profiles and clinical parameters exhibits a quasi-sequential structure due to correlated gene expression patterns and their biological pathway relationships. The inclusion of recurrence-free survival follow-up times introduces a temporal dimension to the prediction task. The gating mechanisms of the LSTM unit allow for the selective retention and forgetting of contextual information, which is advantageous for high-dimensional biomedical data that contain partially imputed values.

In more risky circumstances, the gate that receives input could be trained to add Disease-Free Survival to the state of cells more frequently, and this could represent a powerful signal of recurrence probability. To reveal the hidden state that passes to the following LSTM cell, the result of a gate highlights specific properties. If characteristics such as stage and treatment type are associated with malignant recurrence, they have a significant impact on the underlying state. Based on the hidden features, the model is learned through multiple steps, utilizing features such as disease-free survival and recurrence risk, which are detailed in the algorithm above.

The GSE44001 dataset is downloaded from the NCBI Gene Expression Omnibus (GEO); it consists of clinical and gene expression data from 300 cervical cancer patients [41]. The dataset contains clinical features such as tumor stage, the largest diameter of the tumor, disease-free survival (DFS) in months, and DFS status. Kaplan–Meier survival analysis is performed using the GSE44001 dataset to examine stage-wise variations in survival probabilities among patients, showing the difference in recurrence and mortality risk across tumor stages [42].

To detect long non-coding RNAs (lncRNAs) linked with recurrence, gene annotation is performed using the HUGO Gene Nomenclature Committee (HGNC) and GENCODE databases. The Linear Models for Microarray Data package in R is used to recognize long non-coding RNAs (lncRNAs) between recurrence and non-recurrence groups [43]. The GSE44001 clinical attribute file and gene family information are downloaded through R 4.5.2 packages, merged, and converted into a CSV file containing 29,378 gene IDs and corresponding clinical data for 300 patients. Using GENCODE and HGNC annotations, 249 lncRNA gene signatures are identified. The dataset was quantile-normalized to ensure comparability across samples. A list of long non-coding RNAs, downloaded from Gencode [44], is mapped to the GSE44001 Dataset. The GSE44001 Dataset is quantile-normalized, ensuring that long non-coding RNA samples are matched across samples.

The least absolute shrinkage and selection operator is applied to train the dataset and find the relevant lncRNA [45]. Since it can select a specific group of significant lncRNAs by reducing the number of characteristics by setting the coefficients of fewer significant variables to zero, LASSO is frequently employed for feature selection in gene expression analysis. By using LASSO, the features that are not well matched will be reduced, and important features will be matched well.

The Objective Function for LASSO Regression is:

(17) $$objective\;function\;=\;(\frac{1}{2M}{{(q}_{i}-P_{i}.\alpha )}^{2}+\lambda \sum_{j=1}^{p}\left|\alpha_{j}|\right)$$

$q_{i}—Target\;variable\;for\;a\;patient\;i$.

$P_{i}—Vector\;of\;Gene\;expression\;value$.

α—Coefficient of lncRNA.

M—No. of observation.

p—No. of lncRNA Prediction.

λ—The regularisation variable that regulates the shrinkage rate.

Equation (17) is the objective function of the LASSO Regression, which is used to minimize the prediction error between the actual outcome and the predicted outcome. The penalty term controlled by λ makes the less important features decline toward zero. It performs well.

A 10-fold cross-validation was applied to optimize the model and prevent overfitting [46].

Based on the selected lncRNAs and their LASSO coefficients, individual risk scores are calculated as:

(18) $${Risk\;score}_{j}=\sum_{i\epsilon S}Y_{ij}.\alpha_{j}$$

$Y_{ij}—Expression\;level\;of\;lncRNA\;j\;for\;patient\;i$.

$\alpha_{j}—LASSO\;Coefficient\;for\;lncRNA$.

S—Set of selected lncRNA by LASSO.

Equation (18) is used to find the individual recurrence risk score for each patient, which is calculated as a weighted sum of the expression value for selected RNA. The Positive coefficients indicate that higher expression increases risk and reduces DFS; negative coefficients show a protective effect.

After the analysis, the nine lncRNA gene signatures—*ATXN8OS*, *C5orf60*, *DIO3OS*, *EMX2OS*, *INE1*, *KCNQ1DN*, *KCNQ1OT1*, *LOH12CR2*, and *RFPL1S*—are found to be significant predictors of cervical cancer recurrence [47]. These features were further validated using Cox regression to derive risk scores, which formed the basis for the composite risk assessment model.

## 4. Results

Real-time clinical data are gathered from Shanmuga Hospital, Salem, and the GSE44001 Data is downloaded in the NCBI GEO Database. Finally, common high-risk factors are identified. Clinical high-risk factors are disease-free survival, stage, lymph node metastasis, tumor size, and PV/PR examination. Datasets have been linked to long non-coding RNA Gene expression data using the following procedures. The GENCODE Annotation tool is used to filter out the long non-coding RNA. They are using the GENCODE system to link gene IDs to known long non-coding RNAs (lncRNAs) in order to annotate the GSE44001 expression dataset. Mapped the gene identifiers with only long non-coding RNA from the GSE44001 Dataset. Matching the patients in the original dataset with GSE44001 Clinical attributes through Staging and Recurrence status using the R language can be categorized into reduced risk, Moderate risk, and Elevated risk using the chosen extended non-coding RNA attributes, as determined by LASSO and the selection of significant lncRNAs. Determine a risk value for each individual based on the chosen lncRNAs and the associated overfitting coefficients.

The research builds an LSTM model with 10-fold cross-validation for data preparation and feature selection using RFE. Cross-validation shows a reliable assessment of the model’s performance, and regularization techniques are applied to minimize overfitting. The model is trained, and features are identified to evaluate risk prediction. The integration of clinical data with gene expression profiles enables the system to determine individual risk scores, categorizing patients into three risk groups: reduced, moderate, and elevated risk, based on the selected long non-coding RNAs (lncRNAs) and their associated coefficients.

**Figure 4:**
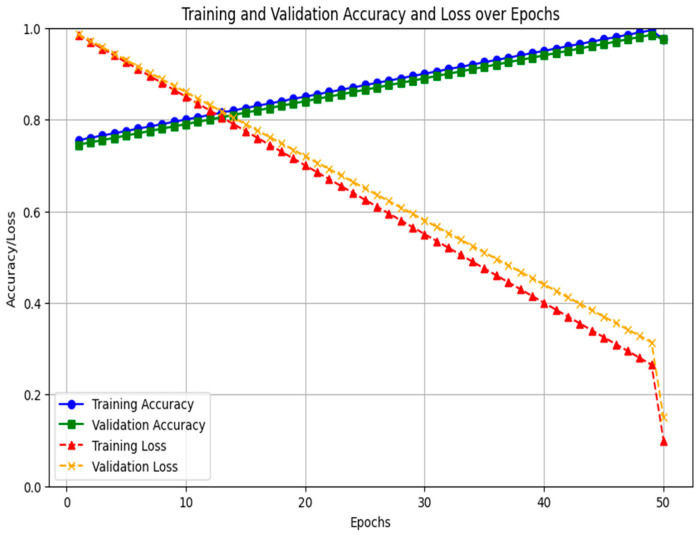
Training and validation accuracy loss.

A two-stage feature selection and validation strategy is implemented to minimize overfitting. Dimensionality is reduced using Random Forest Recursive Feature Elimination (RF-RFE) and Least Absolute Shrinkage and Selection Operator (LASSO) regression, which reduces complexity and identifies the most predictive lncRNA features. This indicates that the number of input variables to the classifier is reduced prior to model training. A recurrent neural network LSTM classifier is trained using Tenfold cross-validation to maintain balanced recurrence status across folds. Hyperparameters were optimized within the cross-validation loop to prevent information leakage, effectively mitigating the risk of overfitting in the high-dimensional, low-sample-size setting. Figure 4 shows the training and validation accuracy loss for 1 to 50 epochs.

**Figure 5:**
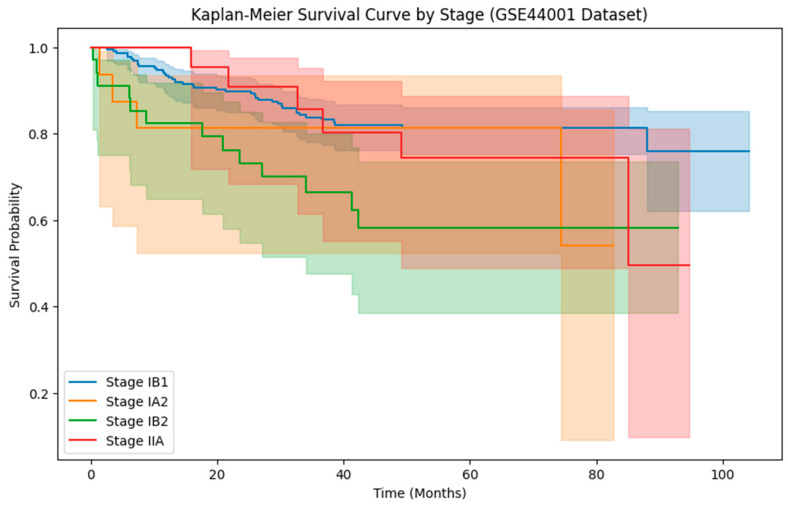
Kaplan Meier survival curve by stages.

Using the GSE44001 dataset’s Kaplan–Meier survival curve by stage, focus on the variations in mortality odds among various tumor stages. The Kaplan–Meier survival curve in Figure 5 illustrates the survival rates of individuals with cervical carcinoma at various stages, including Stage IB1, IA2, IB2, and IIA, over time. Throughout the observed time period, patients at Stage IB1 had the best chance of surviving. In comparison to the remaining phases, the slope for this one is relatively steady, indicating a reduced recurrence. The Stage IA2 survival curve begins similarly to that of Stage IB1, but it displays a more rapid decline in mortality likelihood over time.

In comparison to Stage IB1, this corresponds to a higher recurrence rate. Stage IB2 has a moderate survival probability. Individuals at Stage IIA have the highest risk of recurrence and the sharpest decline in survival likelihood.

Cervical carcinoma that only spreads to the cervix or has minimal dissemination is referred to as the initial stages of cervical cancer. Cervical cancer that has expanded outside of the cervix yet has not spread to other areas is referred to as LACC [48]. Although growth continues to occur within the pelvic area, it is more common in later stages of development. Cervical carcinoma, which has progressed outside the pelvic area to distant organs, is referred to as advanced-stage cervical cancer. It is anticipated that cervical carcinoma is going to be the first cancer to be eradicated by humans [49].

**Figure 6:**
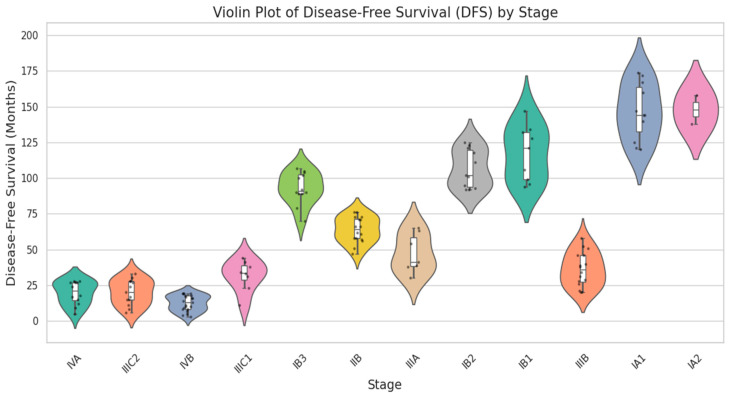
Violin plot for disease-free survival vs. staging categories.

In Figure 6, a Violin plot displays the median and interquartile range (IQR) as white box plots enclosed within each violin, whereas black jittered dots represent specific patient findings. The result reduces the masking impact of high and low values by providing both the raw data range and a statistical summary simultaneously. Stage-related variations in DFS are confirmed by visual inspection, with broader violins in advanced stages indicating greater variation. Since DFS has an irregular distribution, comparisons between groups were conducted using the Kruskal–Wallis test (*p* < 0.001), a nonparametric statistical technique. Real-time data collected from Shanmuga Hospital, Salem, is compared with GSE44001 Data for clinical characteristics. Tumor size and DFS are analyzed. GSE44001 consists of 300 samples of data, with thirty-eight recurrence data and two hundred and sixty-two non-recurrent data.

### Comparison of Common Features

Thus, in the two-dataset comparison, tumor size, disease-free survival (DFS), and recurrence status were identified as the standard variables. To ensure data compatibility and support the integration of the hospital recurrence dataset with the GSE44001 public dataset, a fuzzy matching algorithm is employed based on clinically relevant variables, including tumor size and disease-free survival.

**Figure 7:**
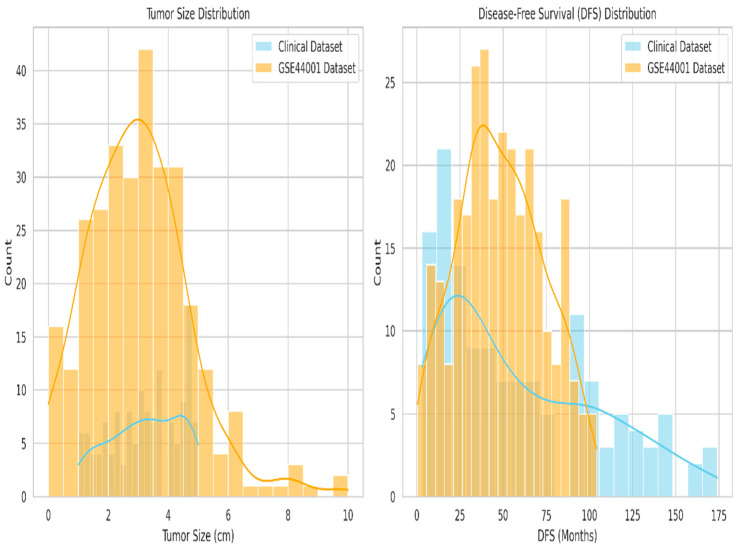
Comparison of common features between tumor size and disease-free survival.

Figure 7 is the comparison of common features in the dataset. After normalizing both features, patients were matched by reducing Euclidean distance, yielding 138 matched pairs in the GSE44001 samples. Strong clinical consistency across datasets was indicated by the average difference in DFS of 8.46 months and the average difference in tumor size of 0.105 cm between matched records.

To statistically validate the comparability of these variables, an independent *t*-test was performed, yielding a non-significant *p*-value of 0.1194 for tumor size, suggesting no significant difference in central tendency. The Kolmogorov–Smirnov test detected a mild distributional shift, *p* = 0.0290, which is expected due to real-world heterogeneity. Cohen’s d values of 0.161 for tumor size and 0.335 for DFS indicate small to moderate effect sizes, thereby supporting the biological alignment of the matched cohorts.

Cohen’s d was used to assess effect size:

(19) $$m=\frac{{\bar{Y}}_{1}-{\bar{Y}}_{2}}{X_{P}}$$

(20) $$X_{p}=\sqrt{\frac{\left(n_{1}-1\right)X_{1}^{2}+(n_{2}-1)X_{2}^{2}}{n_{1}+n_{2}-2}}$$

${\bar{Y}}_{1},{\bar{Y}}_{2}—mean$.

$X_{1},X_{2}—Standard\;deviations\;of\;tumor\;size\;in\;two\;datasets$.

n1,n2—sample size.

$X_{p}—Pooled\;standard\;deviation$.

Cohen’s d is calculated as the standard mean difference between the recurrence and non-recurrence groups in Equation (19). Pooled standard deviation is represented in Equation (20), which is the weighted average of two group variances adjusted by their sample size. Cohen’s d is used to find the effect size between the recurrence and non-recurrence groups. A Cohen’s d of 0.161 for tumor size and 0.335 for DFS indicates small to moderate effect sizes, supporting the biological alignment of the matched cohorts.

Fuzzy matching summaries and statistical validations are presented in Table 4, along with their interpretations. The results of the *t*-test, in which the *p*-value of tumour size comparisons shows less similar differences between the two datasets.

**Figure 8:**
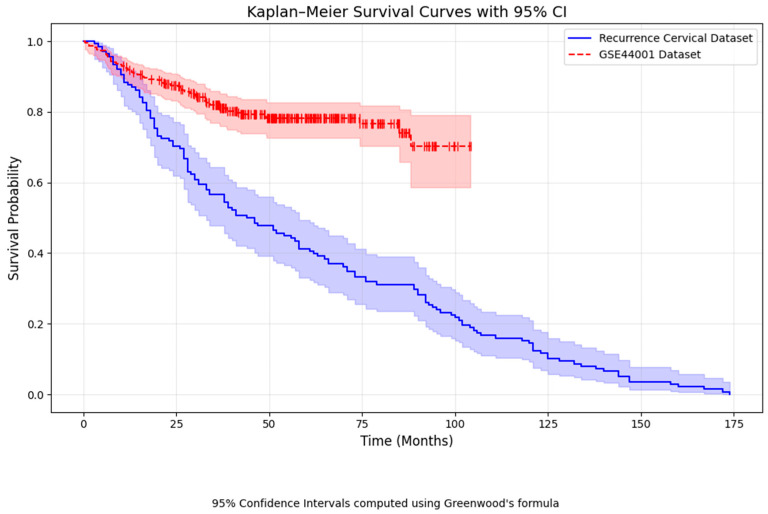
Kaplan Meier survival curve Comparison.

From Figure 8, the Kaplan–Meier survival curve shows that patients in the real-time recurrence cervical cancer dataset have a steep decline in survival, particularly those diagnosed as advanced stages, resulting in a shorter disease-free survival [50]. In comparison, the GSE44001 dataset exhibits longer DFS, whereas the main dataset shows shorter survival times. A chi-square test comparing recurrence and DFS status between the two datasets has a *p*-value of 1.0, indicating no significant difference. From the recurrence of cervical cancer data, the following coefficient table (Table 5) is formed.

The coefficient value for the stage is 0.39, indicating that as the stage increases, the chances of recurrence also increase. A larger tumor size corresponds to a higher hazard ratio, indicating a greater likelihood of recurrence. Long non-coding RNA (lncRNA) profiles from GENCODE are mapped to the GSE44001 dataset and matched based on clinical features, mainly tumor stage and recurrence status. After merging, patients were categorized into three groups: reduced-risk, moderate-risk, and elevated-risk. The contribution of each lncRNA to the risk index is shown by its coefficient. The Positive coefficients indicate that higher expression increases risk and reduces DFS; negative coefficients show a protective effect. Three lncRNA Gene signatures, which have positive coefficients, are *ATXN8OS*, *C5orf60*, and *INE1*. *DIO3OS*, *EMX2OS*, *KCNQ1DN*, *KCNQ1OT1*, *LOH12CR2*, and *RFPL1S* have negative values, which imply a protective effect. Positive expression values reduced the risk and are associated with a greater chance of survival.

**Figure 9:**
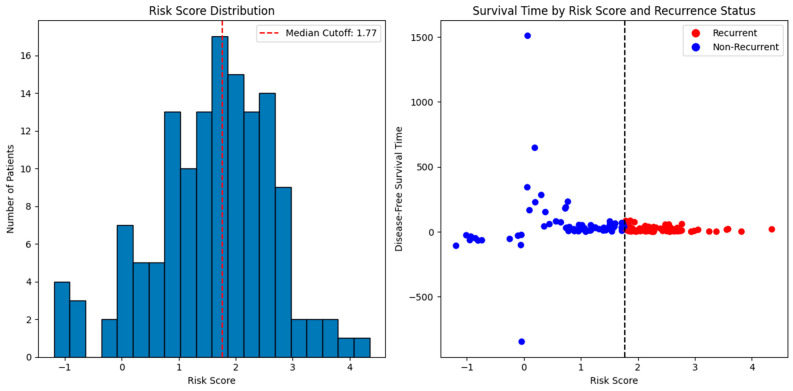
Risk score Distribution and survival time prediction.

Figure 9 shows the distribution of risk scores derived from nine long non-coding RNA (lncRNA) gene signatures. The histogram shows the median risk score (red line), which acts as the cut-off point to classify patients into two groups. The high-risk group is above the median, and the low-risk group is below the median. A scatter plot of disease-free survival time versus risk scores is shown in the same figure. As predicted by the nine-lncRNA prognosis method, shorter survival periods with greater probabilities of recurrence are often associated with higher risk values.

**Figure 10:**
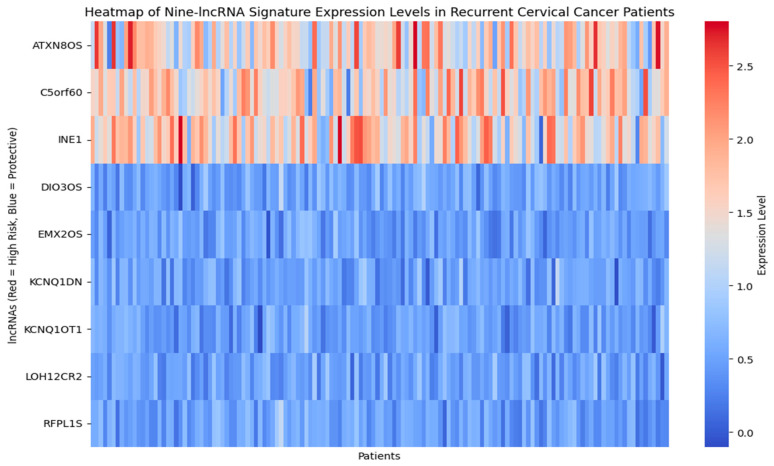
Heatmap of Nine–lncRNA Signature expression level.

**Figure 11:**
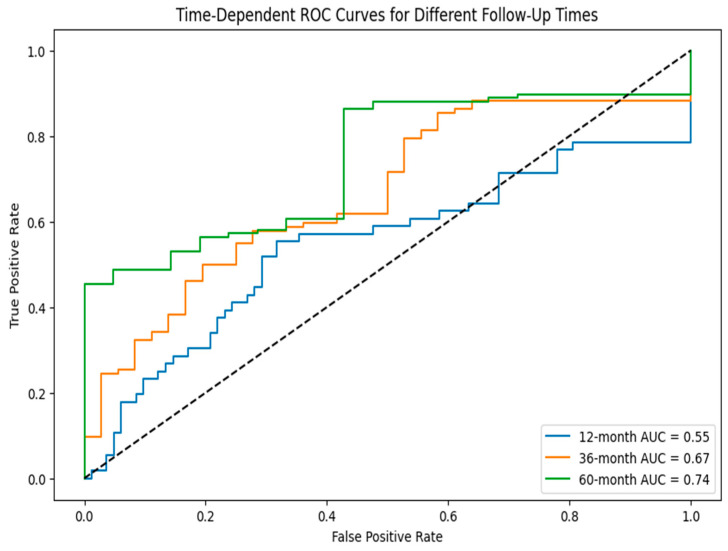
Time-dependent ROC curve for different follow-up times.

Figure 10 presents the heat map of nine long non-coding RNA signature expression levels, showing the stratification of patients into reduced risk, moderate risk, and elevated risk categories through distinct color patterns. Figure 11 shows the time-dependent ROC curves for different follow-up periods, which show the prognostic performance of the nine-lncRNA LSTM model. The model’s predictive accuracy improved over time, with the gradual emergence of prognostic factors due to either slow changes in clinical markers or cumulative effects that become apparent in the medium to long term. According to the time-dependent ROC analysis, the AUC values at 12, 36, and 60 months were 0.55, 0.67, and 0.74, respectively. This trend indicates that the model is more effective at differentiating recurrence risk during extended follow-up periods. The reliability of these estimates is evaluated using 1000 bootstrap resamples, yielding 95% confidence intervals of 0.51–0.60 for 12 months, 0.63–0.71 for 36 months, and 0.70–0.78 for 60 months.

The Chi-square test is a method to confirm that high-risk patients have higher recurrence rates. The results in Table 6 show the relationship between reduced, Moderate, and Elevated risk recurrence rates. The ROC Curve shows the capability of the nine-lncRNA signatures to differentiate between high- and low-risk patients. In the Chi-square test performance, the high-risk group consists of 74 individuals, and the low-risk group consists of 64 patients, as analyzed by their stages. The *p*-value less than 0.05 indicates that patients with an increased risk, as determined by the nine-lncRNA signature, experienced a significantly greater chance of recurrence than patients with low risk, which is supported by this data.

To confirm the robustness of results, additional quality control and statistical validation are performed using the GSE44001 dataset.

**Figure 12:**
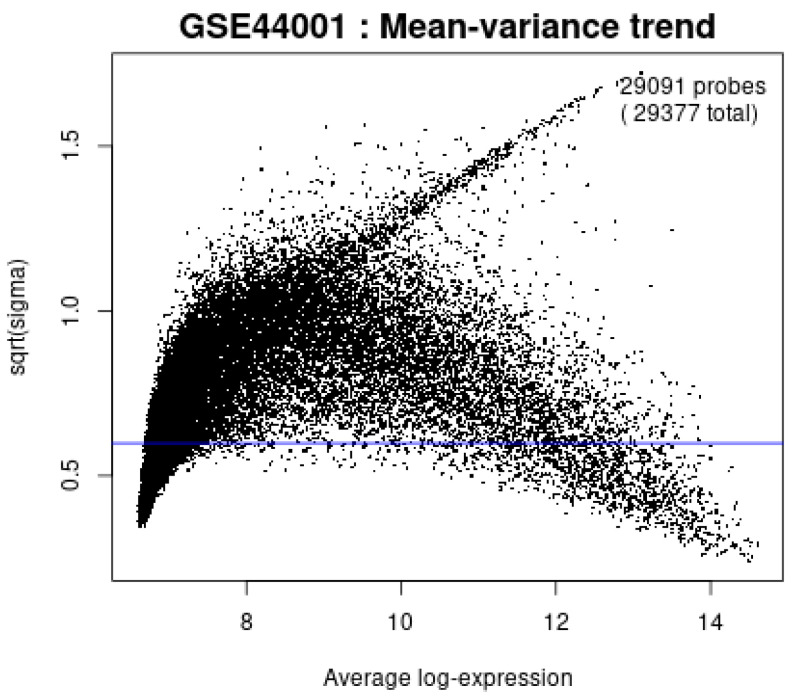
Mean-variance trend plot.

Figure 12 confirms that the mean–variance trend plot confirms the variance of expression values stabilizes after log-transformation, supporting the assumptions of the LIMMA framework for differential expression analysis.

**Figure 13:**
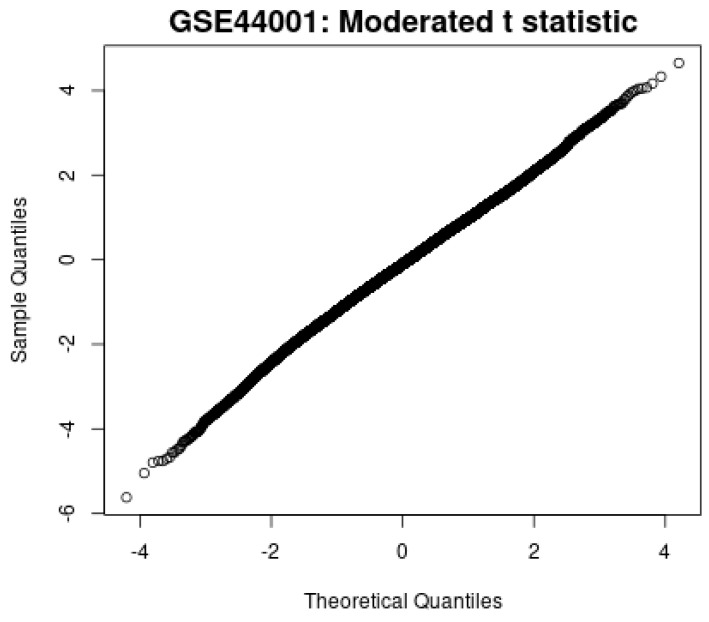
Moderated t-statistic plot.

Second, the moderated t-statistic plot in Figure 13 shows the close alignment between the observed and theoretical quantiles, indicating that the statistical tests follow the expected null distribution and reduce the risk of inflated false positives.

**Figure 14:**
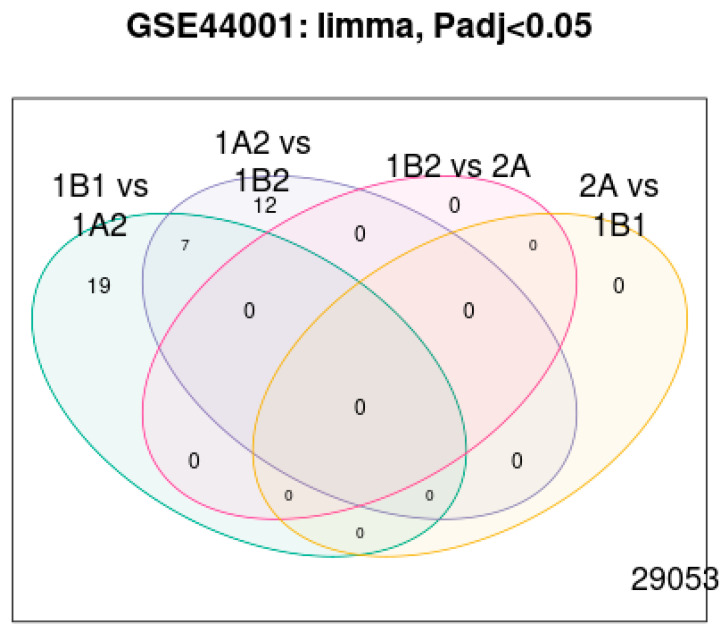
LIMMA Differential expression.

Finally, the LIMMA differential expression Venn diagram in Figure 14 shows the overlaps among differentially expressed lncRNAs across patient subgroups (*p* adj < 0.05). This confirms that the selected lncRNAs are not a single subgroup, but consistently associate with recurrence risk across comparisons.

These analyses provide strong evidence that feature selection and the modeling pipeline are reliable. The real-time dataset is matched with the GSE44001 dataset based on tumor stages. From this analysis, patients with advanced stages of cervical cancer were associated with the ATXN8OS marker, C5orf60 indicator, and INE1 index gene. In contrast, patients diagnosed at earlier stages are linked with the KCNQ1DN marker, LOH12CR2 gauge, RFPL1S value, and KCNQ1OT1 indicator. Patients in moderate stages were primarily associated with the EMX2OS score. This connection between GSE44001 analysis and real-time clinical data validates the robustness of the nine-lncRNA gene signatures, suggesting that they classify patients by recurrence risk.

## 5. Discussion

By combining molecular signatures with clinical features, a nine-lncRNA prognostic model is developed to classify patients into three groups based on reduced, moderate, and elevated recurrence risk. This integrative approach enhances the potential of combining transcriptomic and clinical information in recurrence prediction. This research integrates a real-time cervical cancer recurrence dataset with the publicly available GSE44001 dataset to identify long non-coding RNA signatures. By applying a feature selection framework, the nine-lncRNA signatures are identified in relation to recurrence risk and disease-free survival. Clinical high-risk factors are disease-free survival, stage, lymph node metastasis, tumor size, and PV/PR examination. Results show that three long non-coding RNAs, *ATXN8OS*, *C5orf60*, and *INE1*, are associated with recurrence risk factors. Findings from prior studies indicate that dysregulated lncRNAs, such as *HOTAIR* and *MALAT1*, promote cervical tumor progression and metastasis [51]. lncRNAs such as *DIO3OS*, *EMX2OS*, *KCNQ1DN*, *KCNQ1OT1*, *LOH12CR2*, and *RFPL1S* have been shown to associate with a negative gene signature, which links lncRNA to tumor suppression mechanisms [21]. The Kaplan–Meier and time-dependent ROC curves reinforced the predictive capability of nine-lncRNA, with prediction accuracy improving over more extended follow-up periods. This finding aligns with earlier survival-based lncRNA studies in gynecological cancers, which suggest that extended observation enhances predictive discrimination. Long non-coding RNA signatures have predictive power compared with clinical features, particularly tumor size and stage [52].

*ATXN8OS*, *C5orf60*, and *INE1* risk scores are 28.45, 32.18, and 75.93, indicating a strong correlation with shorter disease-free survival and increased recurrence probability. These results suggest that these lncRNAs act as oncogenic drivers in cervical carcinoma, consistent with previous findings that several lncRNAs promote tumor progression. whereas *DIO3OS*, *KCNQ1DN*, *LOH12CR2*, *RFPL1S*, and *KCNQ1OT1* showed protective effects, correlating with longer progression-free survival. lncRNAs act as either tumor suppressors or oncogenes in cancer development, the mixed predictive scores shown here reflecting the same. Time-dependent ROC analysis validates the model; the reliability of these estimates is evaluated using 1000 bootstrap resamples, yielding 95% confidence intervals of 0.51–0.60 for 12 months, 0.63–0.71 for 36 months, and 0.70–0.78 for 60 months. This supports the nine-lncRNA long-term prognostic value by suggesting the long-term impact of molecular dysregulation. The practical implications of the research are that patients with elevated-risk signatures should undergo closer surveillance and aggressive adjuvant therapy, while low-risk patients can avoid overtreatment. Integrative analysis identified a nine-lncRNA signature with distinct prognostic associations in cervical cancer recurrence as shown in Table 6. This shows that molecular markers complement traditional clinical prognostic factors, improving personalized treatment planning. This research provides new insights, such as the integration of real-world hospital data with GSE44001, which enhances external validation. Limitations include a modest sample size compared to larger population-based cohorts.

In future, we plan to collaborate with additional hospitals as an extension of this research. RNA sequencing data contain inherent batch effects, despite efforts to normalize and preprocess them. Findings of this research demonstrate that a nine-lncRNA prognostic signature derived from both real-time and public datasets stratified cervical cancer patients by recurrence risk. This integrative approach enhances the generalizability of results, bridges clinical and transcriptomic data, and paves the way for future studies and potential translational applications in risk prediction and therapy optimization.

## 6. Conclusions

This Research investigates lncRNA gene signatures associated with cervical cancer recurrence by integrating clinical features with lncRNA expression profiles. Clinical data were matched with the GSE44001 dataset using fuzzy alignment of tumor stage and disease-free survival (DFS), and relevant lncRNAs were identified using the LIMMA framework. Through feature selection and analysis, a nine-lncRNA signature is found as a predictor of recurrence risk based on clinical factors. Among these, INE1, C5orf60, and ATXN8OS were identified as high-risk markers; the other six gene signatures are associated with protective effects. Patients with a considerably shorter DFS and greater recurrence rates were identified as high risk based on this characteristic.

The accuracy of predictions is enhanced by the model’s ability to identify intricate relationships between molecular and clinical variables through the use of an LSTM-based recurrent neural network. These results highlight the potential of lncRNA-driven predictive models for patient categorization. Crucially, the nine-lncRNA analysis provides an important resource for identifying high-risk patients, enabling individualized treatment plans and more stringent monitoring. Future validation in larger, multi-center groups and functional analysis to reveal the biological mechanisms of these lncRNAs is crucial for advancing this signature toward clinical application.

## Figures and Tables

**Table 1:** Recurrence of cervical cancer patient details.

Category	Details
Total patients with recurrence (*n*)	138
Age (years), mean ± SD	49.7 ± 14.6
FIGO 2009 staging	*n* (%)
IVB	21 (15.2%)
IVA	12 (8.7%)
IIIC-2	13 (9.4%)
IIIC-1	8 (5.8%)
IIIB	16 (11.6%)
IIIA	7 (5.1%)
IIB	16 (11.6%)
IB3	12 (8.7%)
IB2	11 (8.0%)
IB-1	9 (6.5%)
IA-2	2 (1.4%)
IA-1	11 (8.0%)
Stages, *n* (%)	
Early	22 (15.9%)
Locally advanced	67 (48.6%)
Advanced	49 (35.5%)
Histological subtypes, *n* (%)	
Squamous cell carcinoma (SCC)	51 (37.0%)
Adenocarcinoma (ADC)	53 (38.4%)
Other	34 (24.6%)

**Table 2:** Statistical table for numeric values in the dataset.

Features	Mean	Median	Standard Deviation
Age	49.65	50.0	14.57
Age of Initial Diagnosis	50.35	52.0	14.89
Post Menopause(years)	7.60	6.0	7.6
Tumor size	3.21	3.34	1.183
DFS months	59.45	45.0	45.75

**Table 3:** Extent of missingness for each clinical and imaging feature.

Feature	Missing (*n*)	Missing (%)
Age	5	0.7%
Age of Initial Diagnosis	6	0.8%
Post Menopause in Years	28	3.8%
Symptoms	10	1.4%
Duration of Symptoms	35	4.7%
Comorbidities	12	1.6%
Comorbidities Details	45	6.1%
Addictive Habits	60	8.1%
PV Examination	15	2.0%
PR Examination	18	2.4%
Primary Lesion—MRI	55	7.4%
Primary Lesion—CT Scan	110	14.9%
HPV Infection	95	12.9%
HPV Vaccination Status	140	18.9%
Smoking	80	10.8%
Chlamydia Infection	65	8.8%
BMI	50	6.8%
Oral Contraceptives Use	90	12.2%
Number of Full-term Pregnancies	25	3.4%
Age at First Full-term Pregnancy	60	8.1%
History	75	10.1%
Tumor Size (cm)	30	4.1%
Lymph Node Metastasis	40	5.4%
Histological Type	15	2.0%
Treatment Type	5	0.7%
FIGO Stage	0	0.0%
Imaging	12	1.6%
DFS (Months)	0	0.0%

**Table 4:** Fuzzy matching summary and statistical validation.

Metric	Value	Interpretation
Number of matched records	138	Successfully matched patients using tumor size and DFS
GSE 44001 Data	299	Full public dataset size
Tumor size difference in average (cm)	0.105	Very close alignment in tumor size
DFS difference average(months)	8.457	Acceptable difference considering real-world variability
*t*-test *p*-value (Tumor Size)	0.1194	No significant difference in tumor size distributions
KS-test *p*-value (Tumor Size)	0.0290	Mild distributional shift detected
Cohen’s d (Tumor Size)	0.161	distributions are broadly similar

**Table 5:** Coefficient table for recurrence of cervical cancer.

Variable	Coef	exp(Coef)	Z	*p*-Value	95% Confidence Interval
Stage	0.39	1.48	13.92	<0.005	(1.40–1.56)
Largest Diameter (cm)	0.17	1.19	2.93	<0.005	(1.06–1.33)

**Table 6:** Estimated Prognosis of Different Biomarkers.

Biomarker	Prognostic Value	Classification	Estimated Prognosis
ATXN8OS Marker	28.45	Elevated Risk	Decreased progression-free period
C5orf60 Indicator	32.18	Elevated Risk	Decreased progression-free period
INE1 Index	75.93	Elevated Risk	Decreased progression-free period
DIO3OS Metric	−45.27	Reduced Risk	Prolonged progression-free period
EMX2OS Score	−1.25	Moderate Risk	Medium progression-free period
KCNQ1DN Marker	−4.87	Low Risk	Prolonged progression-free period
LOH12CR2 Gauge	−0.85	Low Risk	Prolonged progression-free period
RFPL1S Value	−0.62	Low Risk	Prolonged progression-free period
KCNQ1OT1 Indicator	−0.95	Low Risk	Prolonged progression-free period

## Data Availability

The datasets used and analyzed during the current study were collected from Shanmuga Hospital, Salem. The original data presented in the study are openly available in NCBI GEO at GSE44001 accession number.
